# White Matter Abnormalities and Structural Hippocampal Disconnections in Amnestic Mild Cognitive Impairment and Alzheimer’s Disease

**DOI:** 10.1371/journal.pone.0074776

**Published:** 2013-09-27

**Authors:** Jared Rowley, Vladimir Fonov, Ona Wu, Simon Fristed Eskildsen, Dorothee Schoemaker, Liyong Wu, Sara Mohades, Monica Shin, Viviane Sziklas, Laksanun Cheewakriengkrai, Amir Shmuel, Alain Dagher, Serge Gauthier, Pedro Rosa-Neto, for the Alzheimer's Disease Neuroimaging Initiative

**Affiliations:** 1 Translational Neuroimaging Laboratory, McGill Centre for Studies in Aging (MCSA), McGill University, Montreal, Quebec, Canada; 2 Department of Neurology, Xuan Wu Hospital, Capital Medical University, Beijing, China; 3 McConnell Brain Imaging Centre, Montreal Neurological Institute, McGill University, Montreal, Quebec, Canada; 4 Montreal Neurological Institute, McGill University, Montreal, QC, Canada; 5 Athinoula A. Martinos Center for Biomedical Imaging, Charlestown, Massachusetts, United States of America; 6 Center of Functionally Integrative Neuroscience, Aarhus University, Aarhus, Denmark; National Hospital of Utano, Japan

## Abstract

The purpose of this project was to evaluate white matter degeneration and its impact on hippocampal structural connectivity in patients with amnestic mild cognitive impairment, non-amnestic mild cognitive impairment and Alzheimer’s disease. We estimated white matter fractional anisotropy, mean diffusivity and hippocampal structural connectivity in two independent cohorts. The ADNI cohort included 108 subjects [25 cognitively normal, 21 amnestic mild cognitive impairment, 47 non-amnestic mild cognitive impairment and 15 Alzheimer’s disease]. A second cohort included 34 subjects [15 cognitively normal and 19 amnestic mild cognitive impairment] recruited in Montreal. All subjects underwent clinical and neuropsychological assessment in addition to diffusion and T1 MRI. Individual fractional anisotropy and mean diffusivity maps were generated using FSL-DTIfit. In addition, hippocampal structural connectivity maps expressing the probability of connectivity between the hippocampus and cortex were generated using a pipeline based on FSL-probtrackX. Voxel-based group comparison statistics of fractional anisotropy, mean diffusivity and hippocampal structural connectivity were estimated using Tract-Based Spatial Statistics. The proportion of abnormal to total white matter volume was estimated using the total volume of the white matter skeleton. We found that in both cohorts, amnestic mild cognitive impairment patients had 27-29% white matter volume showing higher mean diffusivity but no significant fractional anisotropy abnormalities. No fractional anisotropy or mean diffusivity differences were observed between non-amnestic mild cognitive impairment patients and cognitively normal subjects. Alzheimer’s disease patients had 66.3% of normalized white matter volume with increased mean diffusivity and 54.3% of the white matter had reduced fractional anisotropy. Reduced structural connectivity was found in the hippocampal connections to temporal, inferior parietal, posterior cingulate and frontal regions only in the Alzheimer’s group. The severity of white matter degeneration appears to be higher in advanced clinical stages, supporting the construct that these abnormalities are part of the pathophysiological processes of Alzheimer’s disease.

## Introduction

Alzheimer’s disease (AD) has been conceptualized by a succession of pathophysiological events beginning with progressive extracellular accumulation of amyloid followed by a variety of neurodegenerative changes such as intracellular accumulation of neurofibrillary inclusions, brain atrophy and cell depletion [1]. In AD, neurodegenerative changes (i.e. tau hyperphosphorylation and cell depletion) follows a typical 6-stage topographic pattern starting in the entorhinal cortex, propagating to the limbic cortex and subsequently to the polymodal association cortex [2]. In fact, the asymptomatic AD, mild cognitive impairment (MCI) and dementia stages nearly correspond to the severity of AD neuropathology propagation [2,3].

From pathophysiological perspective, there is a growing consensus that white matter (WM) abnormalities in MCI constitute an integral part of the degenerative process associated with AD pathophysiology. Chen and colleagues proposed that white matter pathology, as measured *in-vivo* in dementia patients, may be a sign of ‘anterograde Wallerian degeneration’, in which gray matter pathology could be preceded by axonal dysfunction [4]. WM structural changes such as myelin breakdown, loss of myelin basic protein [5], neuroinflammation as well as abnormal axonal transport have been recognized as part of AD WM neuropathological features [6-8].

The role of WM degeneration in AD has been explored in vivo with Magnetic Resonance Imaging (MRI; see review [9,10]). Mean diffusivity (MD) and fractional anisotropy (FA) are MRI diffusion tensor imaging (DTI) outcome measures informative of microstructural organization of water in WM compartments. High MD conveys local increase of free water diffusivity in WM, which possibly is linked to reduction in myelin content, axonal depletion or declines on extracellular matrix [11]. Low FA indicates loss of diffusion directionality, which is imposed by abnormal axonal membranes. In fact, post mortem data show a correlation between FA axonal and myelin WM contents [11]. Advances in image processing allow the estimations of WM pathways, which are derived from Bayesian mathematical models (probabilistic tractography) [12]. These techniques provide a metric to estimate the degree in which WM abnormalities disrupt long pathways connecting distinct brain regions. Thus, assessment of WM abnormalities using MRI can expand classic neuropathological approach by estimating WM structural connectivity in major WM pathways [13,14].

MCI due to AD is a condition characterized by objective cognitive deficits which minimally interfere with activities of daily living [15]. Peterson and colleagues outlined a classification of MCI as amnestic mild cognitive impairment (aMCI) and non-amnestic mild cognitive impairment (naMCI), based on the predominance of memory deficits over other cognitive domains [16]. It has been established that local WM disconnections between the entorhinal cortex and hippocampus (i.e. perforant path) are involved in AD and MCI pathophysiology, however large-scale hippocampal WM connectivity has never been systematically assessed in these populations [17,18]. Large-scale hippocampus structural connectivity indicates the severity of disconnections between limbic and polymodal association cortex. Here, we aimed to compare patterns of brain FA and MD abnormalities as well as hippocampal connectivity among aMCI, naMCI and AD individuals. We hypothesized that there will be greater severity of MD and FA abnormalities in AD. In addition, we predict disconnections on large-scale hippocampal WM networks in AD but not in aMCI or naMCI.

## Methods

Two cohorts were analyzed in this study. The first cohort of aMCI and cognitively normal (CN) individuals was recruited at the McGill Centre for Studies in Aging (MCSA cohort) located in Montreal, Quebec, Canada. An independent cohort of CN, MCI, and AD was obtained from Alzheimer’s Disease Neuroimaging Initiative Go /2 (ADNI cohort). Informed written consent was obtained from each subject in accordance with local institutions’ Research Ethic Boards (REB) [19]. The McGill University Research and Ethics committee approved these protocols.

### MCSA cohort data acquisition

The McGill Centre for Studies in Aging (MCSA) staff was responsible for patient recruitment, screening and enrollment in the MCSA cohort. Patients with subjective memory complaints, substantiated by a knowledgeable informant were clinically assessed (SG). Subsequently, patients underwent a full medical, neurological examination and battery of neuropsychological tests including the standard Mini Mental State Examination (MMSE) and Rey Auditory Verbal Learning Test [20]. Diagnosis of MCI was achieved by a consensus in a clinical diagnosis meeting based on the Peterson criteria [16].

Age and gender matched controls enrolled in this study, referred here as CN, were recruited by advertisements in local newspapers. CN exclusion criteria were (1) presence of current or past neurological or psychiatric condition and (2) history of memory complaints. Exclusion criteria for all subjects included a history of psychological problems, intellectual inability, past psycho stimulant drug use or brain vascular lesions on the Fluid attenuation inversion recovery (FLAIR) MRI.

MRI data was acquired on a Siemens 3T Trio MR scanner (Siemens Medical Systems, Erlangen, Germany) using a 32-channel phased-array head coil. Diffusion encoding was achieved using a single-shot spin-echo echo planar sequence with twice-refocused balanced diffusion encoding gradients. High angular resolution reconstruction was acquired with 99 diffusion encoding and 10 resting (b0) directions, 2mm isotropic voxel size, 63 slices, b=1000 s/mm^2^, TE=89ms, TR=8.3s. A 1mm isotropic resolution T1-weighted anatomical scan was also acquired (TR=18ms, TE=10ms, FA=30 degrees). The two datasets were registered using a mutual information based algorithm [21] to remove image misregistration from echo planar induced image shifts and motion. All scans were conducted at the Montreal Neurological Institute.

### ADNI cohort data description

A second dataset used in the preparation of this article was obtained from the Alzheimer’s Disease Neuroimaging Initiative (ADNI) database (adni.loni.ucla.edu). The ADNI was launched in 2003 by the National Institute on Aging (NIA), the National Institute of Biomedical Imaging and Bioengineering (NIBIB), the Food and Drug Administration (FDA), private pharmaceutical companies and non-profit organizations, as a $60 million, 5-year public-private partnership. The primary goal of ADNI has been to test whether serial magnetic resonance imaging (MRI), positron emission tomography (PET), other biological markers, and clinical and neuropsychological assessment can be combined to measure the progression of mild cognitive impairment (MCI) and early Alzheimer’s disease (AD). Determination of sensitive and specific markers of very early AD progression is intended to aid researchers and clinicians to develop new treatments and monitor their effectiveness, as well as lessen the time and cost of clinical trials.

The Principal Investigator of this initiative is Michael W. Weiner, MD, VA Medical Center and University of California – San Francisco. ADNI is the result of efforts of many co-investigators from a broad range of academic institutions and private corporations, and subjects have been recruited from over 50 sites across the U.S. and Canada. The initial goal of ADNI was to recruit 800 subjects but ADNI has been followed by ADNI-GO and ADNI-2. To date these three protocols have recruited over 1500 adults, ages 55 to 90, to participate in the research, consisting of cognitively normal older individuals, people with early or late MCI, and people with early AD. The follow up duration of each group is specified in the protocols for ADNI-1, ADNI-2 and ADNI-GO. Subjects originally recruited for ADNI-1 and ADNI-GO had the option to be followed in ADNI-2. For up-to-date information, see www.adni-info.org.

From the ADNI-GO and ADNI-2 dataset, we selected all participants aged 55 to 90 years of age (inclusive) who had completed, during the course of a single visit, the following clinical, imaging and neuropsychological assessments: T1 MRI, DTI, Mini Mental State Examination (MMSE), Clinical Dementia Rating scale (CDR), Wechsler Memory Scale Logical Memory II, Alzheimer’s disease assessment scale (ADAS)-cog, Rey auditory verbal learning test (RAVLT). Selected individuals were classified as CN, MCI (divided into aMCI, naMCI) and AD, on the basis of clinic-behavioral measures put forth by ADNI.

The data was acquired from 14 centers around the USA and Canada between 2010 and 2012. The scanning parameters were as follows. All diffusion images were scanned on GE 3 tesla scanners. There were 41 diffusion encoding and 5 resting (b0) directions, 1.4mm x 1.4mm x 2.7mm voxel size, b=1000 s/mm^2^. All scans used were already EPI-eddy current corrected by ADNI. T1 scans were acquired on the same GE 3T scanner with a 1.2 mm x 1mm x 1mm voxel size. Further acquisition details are available from ADNI website (ADNI-INFO.org)

### Clinical operational definitions

The criteria for CN included an MMSE score ranging between 24-30 (inclusive), and a CDR score of 0 [22,23]. ADNI2 and ADNIGO define MCI as individuals with subjective memory complaint, an MMSE score between 24-30 (inclusive), objective memory loss as shown on scores on delayed recall of one paragraph from the Wechsler Memory Scale Logical Memory II, a CDR of 0.5, preserved activities of daily living, and the absence of dementia [24,25].

We reclassified ADNI MCI individuals in aMCI or naMCI as defined as 1.5 std. below CN on 30 min auditory verbal learning test (AVLT) delay recall [26]. In addition to the NINCDS/ADRDA criteria for probable AD, mild AD dementia subjects had MMSE scores between 20-26 (inclusive) and a CDR of 1 [27].

### T1 processing

Individual T1 MRIs were skull stripped [28], non-uniformity corrected [29] and registered to MNI152 space nonlinearly [30]. The T1 was then classified into grey matter, white matter, and cerebrospinal fluid with a validated automated classification algorithm [31] and subsequently automatically labeled using Automatic, Nonlinear, Imaging Matching and Anatomical Labeling (ANIMAL) [32]. Individual segmented hippocampus was used as a seed region for the hippocampal structural connectivity (HSC).

### DTI Processing

FA and MD maps were generated using FSL-DTIFIT from the skull-stripped [33] eddy current corrected images in MRI native space.

### HSC Processing

**Figure 1:**
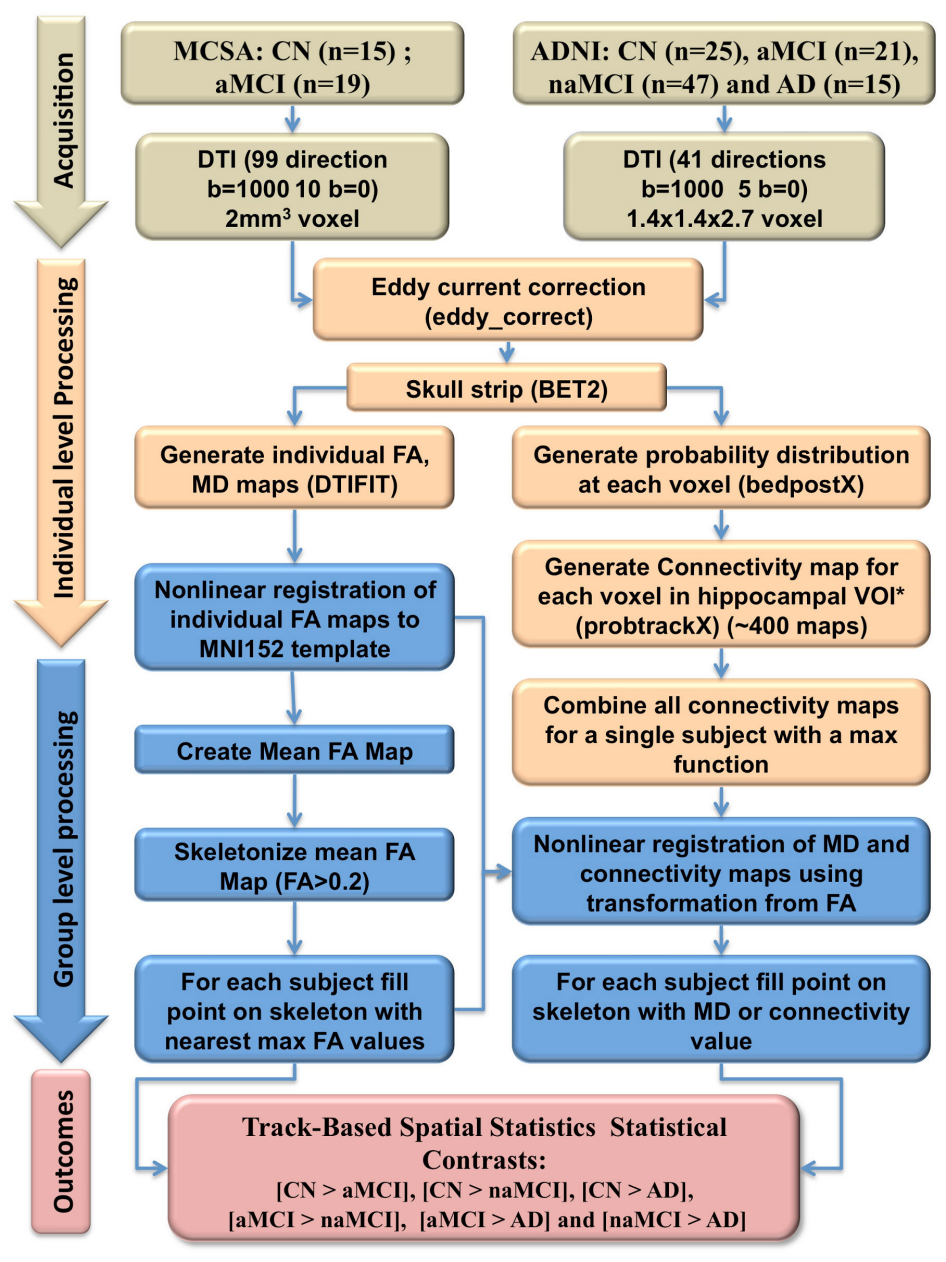
Summary of DTI imaging acquisition, processing and statistical outcomes. Note that DTI acquisition parameters differ for the two cohort, however, the analytical pipeline is identical for the ADNI and MCSA cohorts. Arrows indicate the flow of data through the pipeline. *Hippocampal VOI was estimated using individualized brain segmentations.

HSC maps were generated using a pipeline based on FSL 4.1-FDT (Figure 1). In brief (1), DTI images were skull-stripped using BET2 and Eddy current-corrected [34] ; (2) At each voxel of individual DTI images, a probability distribution of fiber direction was generated using FSL-bedpostx with a maximum of two fiber directions per voxel [35] ; (3) single voxel level probabilistic maps were generated for every voxel of the seed region using FSL-probtrackx (approx. 400 maps per region; see animation in the supplementary materials). These maps represented the probability that any given voxel in the brain was connected to a seed voxel. Finally (4) a single HSC map of the entire hippocampus was computed via the max function derived from the single voxel level maps. Thus a given voxel value in the HSC map represents the likelihood of this voxel being connected to the hippocampus. HSC maps were generated with seed regions in both the left and right hippocampus (see link https://www.youtube.com/watch?v=_ugroLj3iSg).

### DTI Statistics

FA, MD and HSC statistical group differences were created using the track-based spatial statistic tool (TBSS) [36]. Firstly, all FA images were aligned to the MNI 152 standard space [37]. Then a skeleton was created from the mean FA > 0.2. Local maxima of FA images of each subject were then projected onto the skeleton. At each voxel in the skeleton statistical group differences were determined using permutations tests (FSL randomize) [38]. MD and HSC maps were projected onto the same skeletons to determine statistical group differences. The statistical results were thickened to allow better visualization of group differences. Correction for multiple corrections was estimated using family wise error (FWE) and the threshold-free cluster enhancement (TFCE) option [38].

Global averages for FA, MD and HSC were calculated for each subject using the WM skeleton as a region of interest (ROI). In brief, for each statistical contrast comparing two groups, an FA TBSS skeleton was generated by using voxels greater than 0.2. The volume of FA skeleton was then calculated. The proportion of abnormal voxels was computed as the ratio between the volume of voxels showing statistical differences between groups and the total skeleton volume. This strategy intended to avoid the contamination grey matter signal. Regional values of FA, MD and HSC were calculated for each subject using masks based on statistical significant voxels derived from various contrast of interest. To estimate the normalized MD or FA volumes, we computed the ratio between the volume of abnormal voxels when compared to CN as defined by TBSS (corrected p <0.05) and the total WM skeleton volume.

In addition, in order to compare the magnitude of change across patient populations, FA, MD and HSC absolute scores were transformed as z-scores. Transformed FA, MD and HSC values were compared across groups using one-way-ANOVA.

## Results

### Demographics

In the MCSA cohort an unpaired one-tailed t-test showed that CN and aMCI groups did not differ in terms of gender, age, and educations (Table 1a). As expected, MMSE, RAVLT, and APOE status was different between groups.

**Table 1:** Summary of Demographic and memory scores for all groups in the ADNI and MCSA cohorts.

	**MCSACohort**		**ADNI Cohort**			
	**CN**	**aMCI**	**CN**	**aMCI**	**naMCI**	**AD**
N	15	19	25	21	47	15
Gender (M/F)	7/8	8/11	13/12	12/9	29/18	9/6
Age (years)	67±5.7	71.6±8.2	73.3±5.5	73.3±6.9	73.4±7.7	75.7±11.3
Weight (kg)	76±12.6	64.9±12.4	79.9±14.4	76.3±11.6	79.3±13.5	76±15.9
Education (years)	15.3±2.9	14.11±3.4	16.2±2.7	16.3±3	16.2±2.6	16±2.8
MMSE	29.2±1.1	25.9±2.4*	28.9±1.5^b,d^	27.7±1.3^a,d^	28.2±1.5^d^	23.3±1.8^a,b,c^
RAVALT/AVLT	9±2.1	4±3*	12.9±1.6^b,d^	6.7±1.4^a,c^	12.6±1.7^d^	5.4±4.3^a,c^
APOE (carrier/n)	1/8	7/10	4/13	6/10	15/41	4/6

^*^ *t-test in the MCSA cohort revealed significant difference (p<0.05*).

One-way ANOVA Post-hoc Tukey HSD tests are indicated by abbreviations. *^a^*p<0.05, when compared to CN; *^b^*p<0.05, when compared to aMCI; *^c^*p<0.05, when compared to naMCI; *^d^*p<0.05, when compared to AD.

In the ADNI cohort a 1-way ANOVA of the ADNI demographics showed the CN, aMCI, naMCI and AD groups did not differ in terms of gender, age, and education (table 1b). Post-hoc comparisons using Tukey’s HSD indicated significant differences in MMSE, AVLT, and APOE status. There was no demographic difference between the aMCI group and the naMCI group other than the AVLT score.

### DTI outcome measures

**Figure 2:**
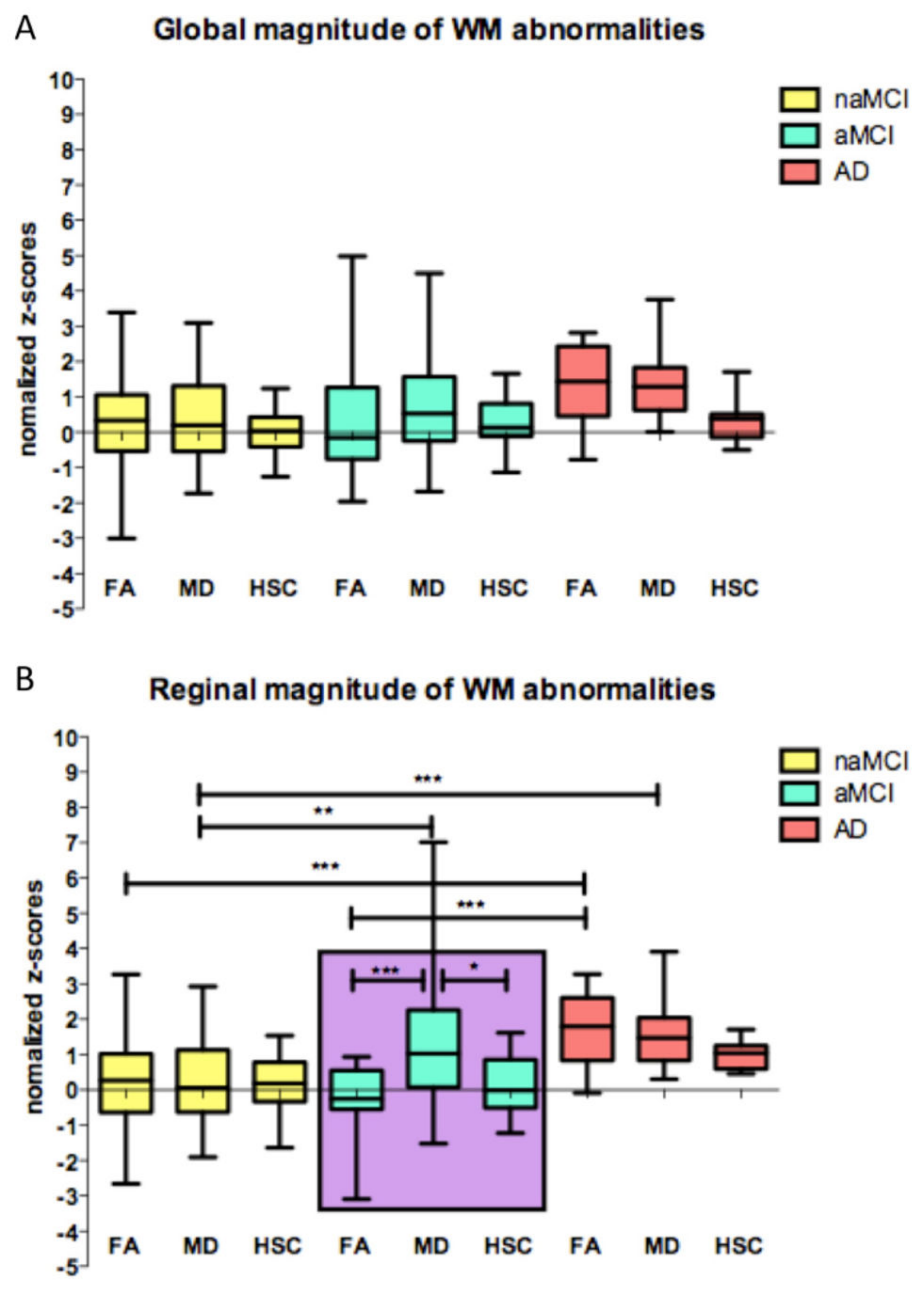
Global and regional magnitude of WM abnormalities in patient population. A shows that magnitude of global MD, FA and HSC abnormalities does not differ among patient groups (One way ANOVA (p=0.0018; F=3.212; df=8). In contrast, regional MD abnormalities predominates in aMCI (purple box) while FA abnormalities arises in AD (Figure 2B -- one way ANOVA (p<0.0001; F=7.74; df=8). Student-Newman-Keuls Post-hoc test ***, p=0.001; **,p=0.01.

**Figure 3:**
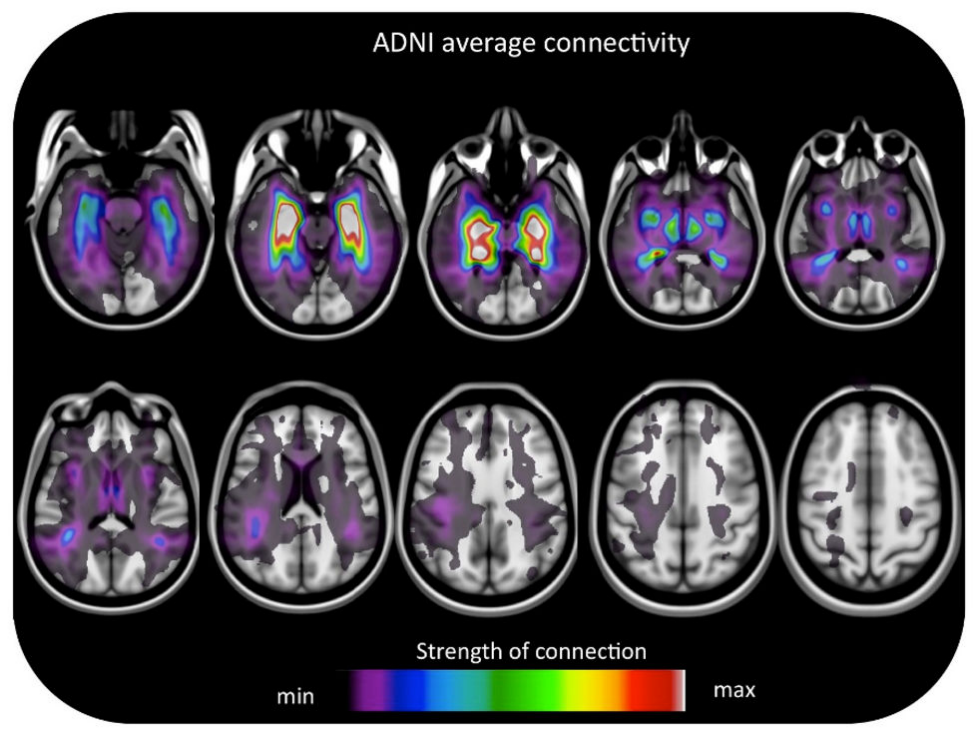
Hippocampal structural connectivity. Average maps expressing the hippocampal to brain structural connectivity for the CN group.

Global and regional FA and MD z-score values are represented in Figure 2A and 2B. Average FA and MD were significantly different between cohorts (FA CN_MCSA_=0.41 vs CN_ADNI_=0.36). The magnitude of global MD, FA and HSC abnormalities does not differ among patient groups (Figure 2A). In addition, while regional MD abnormalities predominate in aMCI, FA abnormalities are higher in AD (Figure 2B). Average HSC obtained in the CN, depicting patterns of hippocampal connectivity known from experiments obtained in post mortem tissue, such as projections to the entire temporal neocortex as well as to cingulate cortex as well as associative parietal occipital and frontal cortices (Figure 3).

### DTI group differences

**Figure 4:**
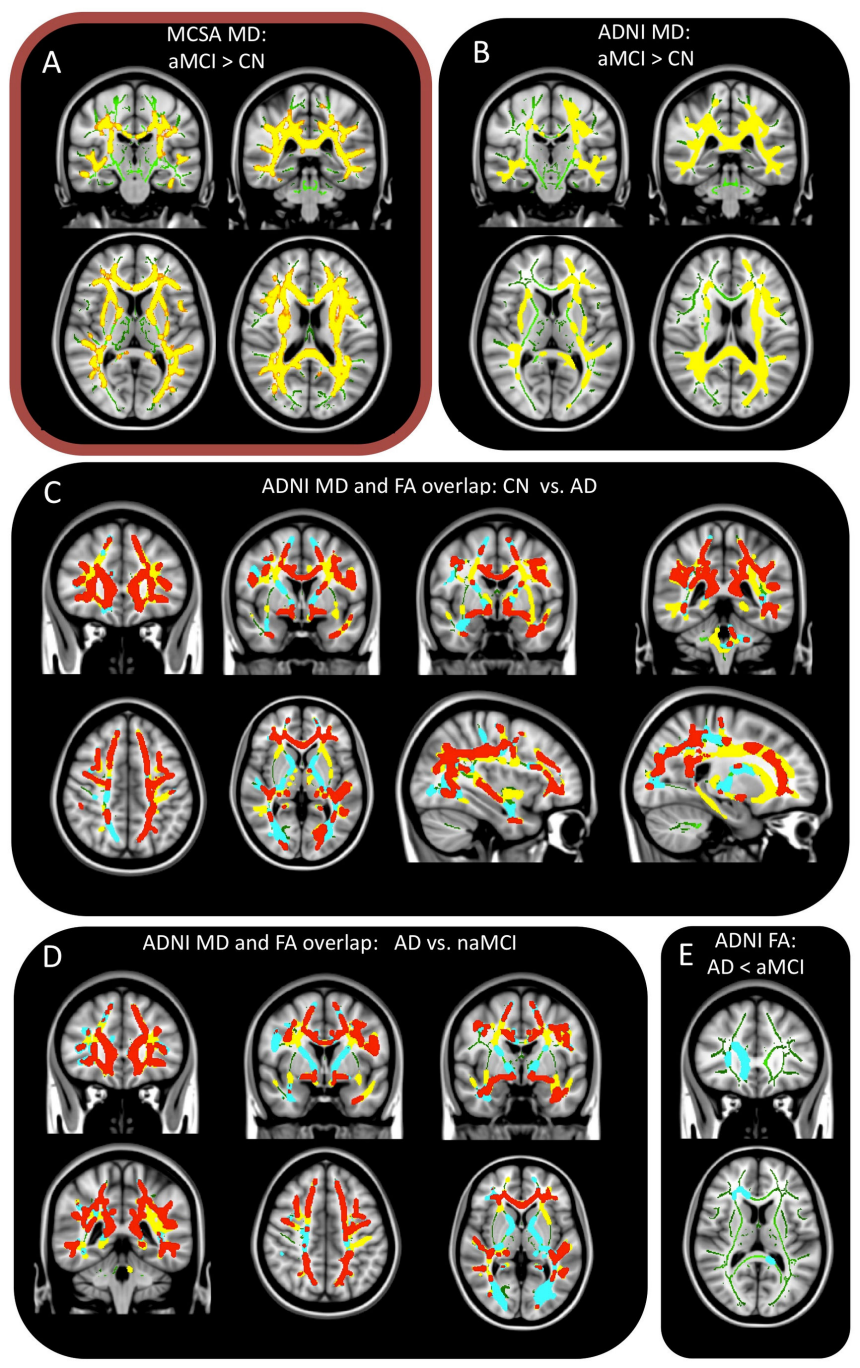
FA and MD group statistics. All parts display voxels showing abnormal FA and MD on TBSS skeleton. Statistically significant group differences of MD (yellow), FA (blue) are shown. A shows the aMCI>CN contrast for the MCSA cohort while B-E show contrasts from the ADNI cohort. Notice C and D show overlap of MD and FA in red. Contrasts not shown were not significant.

In the MCSA aMCI cohort, 29% of estimated WM had significantly elevated MD compared to the CN group. Corpus callosum, arcuate, uncinate, superior and inferior longitudinal fascicles were affected (Figure 4A). Voxels with abnormal MD in aMCI were on average 7.1% higher than CN. Global WM MD was elevated 5.5% in aMCI (p=0.003) compared to CN. None of the clusters of reduced FA were significant after correcting for multiple comparisons. HSC revealed no hippocampal connectivity abnormalities in aMCI.

In the ADNI cohort (Figure 4B) aMCI (aMCI>CN) showed abnormal mean diffusivity in 27.6% of estimated WM, particularly on the corpus callosum, splenium, superior and inferior longitudinal fascicles, and arcuate fascicles. These abnormal voxels had an average of 5.8% higher MD than CN. Global WM MD was elevated 4.1% in aMCI compared to CN (p=0.039) (Table 2). Similarly to the MCSA cohort, ADNI aMCI had no significant declines in FA. No WM abnormalities were observed in the reverse statistical comparison [aMCI< CN]. No hippocampal connectivity abnormalities were observed in aMCI.

**Table 2:** Summary of magnitude of global WM differences across group contrasts.

	**MCSA Cohort**					**ADNI Cohort**				
	**FA**	**MD**		**HSC**		**FA**	**MD**		**HSC**	
CN vs. aMCI	2.73	5.47*		12.23		2.45	4.09*		7.65	
CN vs. naMCI						1.74	2.40		1.78	
CN vs. AD						7.88*	7.69*		9.17	
naMCI vs. aMCI						0.70	1.65		5.97	
naMCI vs. AD						6.04*	5.16*		7.52	
aMCI vs. AD						5.3*		3.45*		1.65

Both MCSA and ADNI cohort indicate MD abnormalities on aMCI. FA abnormalities are present in AD groups. *p<0.05

In naMCI, part of ADNI cohort, neither FA nor MD values differed from CN.

The **AD** patients in the ADNI cohort (Figure 4C) showed abnormally elevated MD in 66.3% of estimated WM compared to CN. These abnormalities were observed particularly in the genu and splenium of the corpus callosum, uncinate, superior and inferior longitudinal fascicles and cingulate bundle. Abnormal voxels in these pathways had an average of 8.5% higher MD than CN. Global WM MD was elevated 7.7% in AD (p=0.0001) (Table 2). In addition, FA was abnormally low in 54.3% of the estimated WM. In these abnormal voxels, FA was 11.3% lower than CN. Interestingly, 75% of WM regions with abnormally lower FA also had abnormally higher MD. Global WM FA was reduced by 7.9% (p=0.0004) (Table 2). No FA elevations or MD declines were observed in AD.

**Figure 5:**
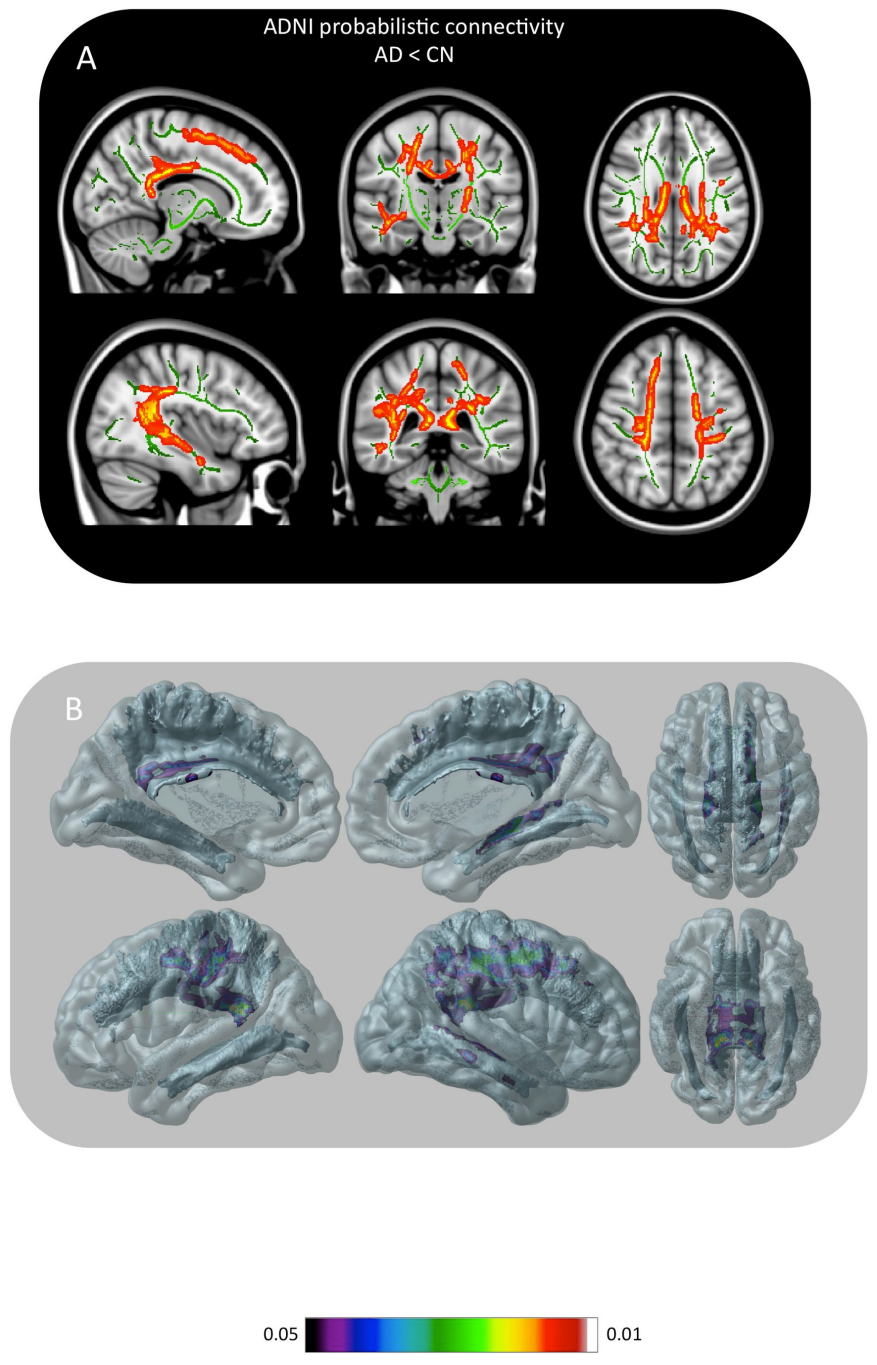
Hippocampal structural connectivity group statistics. **Part A** displays voxels showing reduction of hippocampal connectivity on the TBSS skeleton [AD<CN] after correction from multiple comparisons. Note the reduction in hippocampal connectivity on angular bundle, fornix, superior longitudinal, inferior longitudinal, cingulate, uncinate and arcuate fascicles. **Part B** shows areas of reduced connectivity in AD projected on WM pathways on a transparent brain surface space highlighting regions with reduced connectivity (See 3D animation of this image at http://www.youtube.com/watch?v=LuRgO0I4TZU).

HSC showed reduced hippocampal connectivity (Figure 5A and 5B) in the temporal lobe (angular bundle, inferior longitudinal and uncinate fascicles), limbic projections (cingulate bundle and fornix), inferior parietal cortex (arcuate fascicles) and frontal (inferior occipitofrontal and superior longitudinal fascicles) in AD patients compared to CN (Figure 5; see link https://www.youtube.com/watch?v=LuRgO0I4TZU). All other contrasts were not significant after correcting for multiple comparisons.

The contrasts between **AD and naMCI** patients of the ADNI cohort revealed abnormally high MD in 54% of AD estimated WMs (Figure 4D). Abnormal estimated WM in AD had an average of 7% higher MD than naMCI. Global WM MD was elevated 5.2% in AD as compared to naMCI (p=0.001) (Table 2). In comparison with naMCI, AD patients had abnormally reduced FA in 57.7% of the estimated WM. These abnormal voxels had an average of 10.6% lower FA in AD in comparison with naMCI. Global WM FA was reduced by 6% (p=0.003) in AD in comparison with naMCI (Table 2).

The **AD to aMCI** contrast of the ADNI cohort (Figure 4A) revealed no abnormal MD voxels after correcting for multiple comparisons. Global WM MD was elevated 3.5% in AD as compared to naMCI (p=0.047), however this was just barely significant. FA was abnormal in 0.34% of the normalized WM voxels. Abnormal voxels had 12.5% lower FA than CN. Global WM FA was reduced by 5.3% (p=0.03) (Table 2).

Both FA and MD values in ADNI cohort, showed no abnormalities in naMCI (aMCI vs. naMCI) after correcting for multiple comparisons.

## Discussion

Brain WM forms the backbone of a large network connecting multiple segregated cortical regions, which occupies nearly as much of brain volume as the gray matter [39]. There is a growing body of the literature suggesting active WM degeneration as part of the repertoire of pathophysiological processes underlying AD. Although somehow neglected, the impact of WM abnormalities in AD has been reported to be large [4,11,14].

Here, we report increased MD (6-7%) occurring in approximately 28% of estimated WM and no FA declines in aMCI. In contrast, the WM of AD individuals had slightly higher MD (8.5%) and lower FA (11.3%) affecting a larger proportion (66%) of estimated WM. The magnitudes of MD or FA group differences reported here are above expected variability as revealed by previous DTI test-retest studies [40]. In addition, we report, for the first time, brain areas disconnected from the hippocampus due to WM abnormalities in AD patients using FSL’s probabilistic tractography method [12]. This technique provides the likelihood of connectivity between the hippocampus and any given brain region. Our results support the hypothesis that AD brain undergoes a progressive WM degeneration characterized firstly by increased MD, followed by declines in FA and reduction of hippocampal connectivity.

The modest but significant increase of MD found in aMCI (both MCSA and ADNI cohorts) supports the construct that increased WM water diffusivity constitutes an early neurodegenerative event associated to AD pathophysiological processes. In fact, high MD has been frequently reported in aMCI populations [41-49]. In contrast using TBSS, Agosta and colleagues only found widespread changes in axial diffusivity but no significant MD differences in 15 aMCI individuals, however, these disparities could be explained by methodological issues such as MRI magnetic strength as well as low in-plane resolution [47,48,50].

Absence of FA reduction in aMCI, as reported here, is consistent with previous studies using similar methodology (i.e. same MCI inclusion criteria, 3T acquisitions and strict TBSS analysis) [41,42,50-52]. However, FA declines in MCI have been previously reported using VOI and voxel-based techniques [43,45,46,53-59]. Such discrepancy might be associated with low sample size, diagnostic criteria for MCI, analytical methods (VOI vs. voxel-based) or correction for multiple comparisons (no correction, FDR, random field).

At least two previous studies focusing on FA and MD abnormalities in naMCI provide conflicting results [42,60]. In fact, older age and the presence of multiple pathologies could account for these conflicting results [60].

The two-fold increase of estimated WM showing increased MD in demented patients (as compared to aMCI) supports the hypothesis that WM pathology also progresses in AD. Higher estimated WM showing lower FA in demented individuals in comparison with aMCI or naMCI further corroborates WM progressive degeneration in AD. Reduced FA in AD has been previously reported using VOI and voxel-based techniques [52,54,61-64]. [56,57,65-68] In fact, progression of WM abnormalities has been previously suggested by cross sectional and longitudinal DTI studies [41,42,51,69]. As a whole, these findings suggest increased MD as an early WM degenerative event in AD.

In post-mortem tissue, increase of tissue water diffusivity, as measured as m^2^s^-1^, might occur due the reduction of tissue barriers imposed by various causes (i.e. tissue atrophy, neuroinflammation), however empirical evidence supporting this claim is undermined by the effects of tissue fixation typically utilized in post-mortem / DTI correlation studies [11]. Possibly increased MD in aMCI might represent an early event of WM degeneration present in AD pathophysiology secondary to the reduction of brain extracellular matrix and shrinkage of WM tissue in preclinical AD [70,71].

In addition to the axonal degeneration originating due to death of cortical cell lesions, recent advances in pathophysiological mechanisms underlying FA and MD abnormalities described in AD can be partially attributable to independent WM neurodegeneration. A growing body of literature suggests that WM DTI abnormalities might be secondary to neuroinflammatory factors [72]. Corroborating this hypothesis, imaging studies with PET and molecular imaging agents show an increase of microglia activation and astrocytosis in the WM of aMCI [73-75]. In addition, it is noteworthy that ventricular enlargement (a surrogate of WM atrophy) is able to accurately distinguish between MCI and AD and has been proposed as tool for measuring disease progression in the short term [76].

We also demonstrated hippocampal WM connectivity abnormalities in AD dementia. Hippocampal connectivity abnormalities affected hippocampal connections predominantly to the temporal, parietal, occipital and frontal polymodal associative areas (Figure 5). The structural connectivity outcome measure described here (HSC) represents the probability of a given brain area to be connected with the hippocampal seed region. The hippocampal seed typically encompasses all cornus amonius (CA) sectors, the dentate gyrus and subiculum, since the limits between these areas remains below the resolution of the present DTI acquisition. For example, connectivity declines observed in the inferior occipitofrontal and superior longitudinal fascicles possibly reflect hippocampal connectivity declines mediated via subiculum, which constitutes a critical hub between the hippocampal system and the cortex [77-82]. WM areas with significant decline of probability to be connected to the hippocampus were interpreted as depleted from normal hippocampal connectivity.

The results from our HSC CN maps are in excellent agreement with the post-mortem data describing hippocampal connectivity [83,84]. Individual connectivity maps generated by this study in CN captured the classical reciprocal connections between the hippocampal system and the temporal, cingulate, inferior parietal or frontal cortices. Since the nature of DTI does not permit the inference of directionality, one cannot discriminate between hippocampal-petal and hippocampal-fugal fibers. Areas with reduced structural connectivity revealed by the HSC technique in AD was consistent with functional disconnections frequently reported by the literature. For example, reduced hippocampal connectivity in the cingulate bundle might explain functional disconnection reported between the hippocampus and the posterior cingulate / precuneus frequently described by numerous AD rsfMRI studies [85,86]. Moreover, the WM connectivity reduction of the arcuate fascicles, inferior longitudinal, uncinate fascicles and superior longitudinal fascicles is a possible mechanism underling the [^18^F]FDG signature of AD (hypometabolism in the posterior cingulate, inferior parietal, temporal and prefrontal cortices) [87-89]. Hippocampal WM disconnections as described here corroborate the theoretical framework that emphasizes cortical disconnections as a key feature of AD [90]. In fact, neuropathological, functional neuroimaging and neuropsychological evidence indicate WM disconnections as a pathophysiological mechanism involved in AD.

The relative integrity of hippocampal connections in aMCI or naMCI, as predicted in our hypothesis, is supported by histopathology evidence showing tangle pathology and WM disconnection affecting predominantly transentorhinal projections to the dentate gyrus (perforant path) in aMCI, while projections from the hippocampus and subiculum and the rest of entorhinal cortex are affected in more advanced stages of the disease [2]. Possibly, a seed point in the transentorhinal cortex could better capture perforant path depletion in MCI stage, however transentorhinal connectivity is beyond the scope of this specific study.

### Limitations

Some methodological issues limit the interpretations of the present study. Since this is a cross sectional study, inferences regarding progression of WM pathology in the spectrum of AD clinical manifestations are speculative. The hypothesis posing MD increases as an early WM AD pathophysiology change followed by FA declines should be assessed by appropriate longitudinal studies.

Vascular pathology is certainly a potential confounder in all studies of this nature, since vascular insults and small vessel disease constitute a frequent finding in the MRI of elderly populations. For example, white matter intensities detected in T2 or FLAIR images may have an impact on various DTI outcome measures. However, since all patients recruited in this study, had Hachinski scores lower than 4, the impact of these lesions are unlikely to be clinically significant. In contrast the impact of vascular pathology on DTI outcomes is always a limitation for dementia studies since Hachinski score < 4 does not preclude vascular lesions. Particularly on the MCSA cohort, the presence of white matter intensities was minimal and monitored with 3D FLAIR MRI. Since the results obtained from the MCSA and ADNI cohorts were identical, it seems that vascular pathology affects these two populations in a similar fashion.

Although ADNI provides a powerful database for AD research, the drawback of utilizing DTI acquisitions acquired in 14 different scanners might represent a limitation for this study. While there were large efforts taken to cross-validate MRI scanners, multiple scanners acquisition is an undeniable confounding factor. Although, using the same analytical pipeline, the MCSA cohort yielded higher FA and MD control values in comparison with the same outcome measures from ADNI cohort. High FA and MD values on the MCSA cohort were obtained due to higher signal to noise ratio from the utilization a 32-channel head coil as well as 99 diffusion directions.

Regarding statistical analysis, TBSS is a conservative but extensively utilized method to compare WM change in numerous experimental populations [42,91,92]. Particularly in the case of this study, the results using voxel-based non-parametric statistics provide similar results to TBSS (data not shown). Analytical protocols can potentially constitute a bias particularly for those studies utilizing voxel-based parametric statistical analysis without correcting for multiple comparisons.

In conclusion, we found in aMCI WM abnormalities are characterized by high MD, which are possibly secondary to brain inflammatory changes or WM axons or myelin content depletion. Furthermore, the concomitance of MD and FA abnormalities observed in AD suggests higher degree of WM microstructural lesion, which impacts in large-scale brain structural connectivity. Further longitudinal studies are necessary to corroborate whether a progression of WM disease occurs in the spectrum of clinical manifestation of dementia.
