# Correction: White Matter Abnormalities and Structural Hippocampal Disconnections in Amnestic Mild Cognitive Impairment and Alzheimer’s Disease

**DOI:** 10.1371/annotation/c1e8aa6c-5e8a-4938-a0ed-1a7dc5ead757

**Published:** 2013-12-31

**Authors:** Jared Rowley, Vladimir Fonov, Ona Wu, Simon Fristed Eskildsen, Dorothee Schoemaker, Liyong Wu, Sara Mohades, Monica Shin, Viviane Sziklas, Laksanun Cheewakriengkrai, Amir Shmuel, Alain Dagher, Serge Gauthier, Pedro Rosa-Neto, for the Alzheimer's Disease Neuroimaging Initiative

The following line was omitted from the Funding statement: "Industry Canada MNI center of excellence in commercialization and research grant awarded to AD and AS." 

